# Association of vaccine awareness and confidence on the influenza vaccination status of Al Ahsa, Saudi Arabia residents

**DOI:** 10.1080/21645515.2020.1855954

**Published:** 2021-01-30

**Authors:** Yasser Taher Al Hassan, Eduardo L. Fabella, Edric D. Estrella, Hassan Abdulfatah Al Ramadan, Ahmed Mansour Al Rajeh, Fatimah Hassan Al Saleh

**Affiliations:** a Al Ahsa Public Health Directorate, Ministry of Health, Kingdom of Saudi Arabia; b College of Applied Medical Sciences, King Faisal University, Al Ahsa, Kingdom of Saudi Arabia

**Keywords:** Influenza vaccine, awareness, confidence, adult immunization, communicable diseases

## Abstract

While the Saudi Arabian Ministry of Health has made seasonal influenza vaccination available for several years, there remains a scarcity of vaccination coverage reports outside of the capital city. Understanding factors that affect vaccine uptake is important in developing strategies to improve coverage. This analytic cross-sectional study utilized data from 1377 adult residents randomly selected through a multi-stage sampling procedure from the three administrative sectors of Al Ahsa, Saudi Arabia. Estimates of influenza vaccine coverage were determined for various demographic groups. Logistic regression was applied to determine the associations among the respondents’ awareness on influenza vaccination services, their confidence on the influenza vaccine efficacy and safety and their vaccination status. The estimated influenza vaccination coverage was 44.15% (95% CI = 41.55; 46.79). The proportion of residents who received the influenza vaccine differed between demographic groups. Al Ahsa residents who were aware that influenza vaccine is available and those who were aware that it is available for free were 2.7 and 6.3 times more likely to be immunized (*p* < .001) compared to those who were unaware. Residents who were confident that it is effective in preventing influenza and its complications were 3.6 times more like to be vaccinated while those who were confident that the vaccine is safe were 4.5 times more likely to be immunized (*p* < .001). Seasonal influenza coverage in Al Ahsa remains low despite the availability of free immunization in the government health facilities. Awareness about vaccine availability and confidence in vaccine efficacy and safety were important determinants of vaccination status.

## Introduction

Influenza is a highly contagious viral infection that is transmitted from person to person.^1^ The illness caused by the influenza virus varies from mild respiratory symptoms to severe illness requiring hospitalization and resulting in deaths.^2^

It is estimated that there are 3 to 5 million severe cases of influenza occurring every year. Annual influenza deaths were previously estimated to be as much as 250,000 to 500,000 but the World Health Organization (WHO) has recently raised the estimates to 290,000 to 650,000.^2–5^

Influenza Type A and Type B are responsible for seasonal influenza.^6^ Seasonal influenza rapidly spreads among individuals of any age group with a global attack rate of 5–10% for adults and 20–30% for children.^3^ The incidence of seasonal influenza is highest in the winter months in temperate regions and peaks during rainy season in tropical areas.^7^ Together with Respiratory Syncytial Virus (RSV), influenza virus is associated with majority of cases of acute lower respiratory infection in children and adults.^8^

Influenza can affect any individual but children below 60 months, adults above 65, pregnant women, individuals with chronic medical conditions, and individuals who have immunosuppressive conditions are at greater risk of severe disease. In addition, health care workers are at high risk of acquiring the influenza virus from their patients.^2^

Influenza deaths most commonly occur among the very young, the elderly and the chronically ill.^9^ Adults above the age of 65 are most vulnerable to complications such as cardiovascular disease and respiratory failure.^1^ According to the United States Centers for Disease Control and Prevention (US CDC), the highest deaths are among people who are 75 years or older and in the world’s poorest regions.^10^

Additionally, seasonal influenza infection during pregnancy is linked to higher risk of morbidity and mortality for both the mother and the child.^11^

Vaccination using an inactive form of the virus has been available for 60 years. Annual vaccination against influenza is considered as the most important strategy to prevent influenza and reduce the burden of this disease.^6,12^ It is also known to decrease influenza hospitalizations, complications and death. Moreover, high immunization coverage is seen as a strategy for prevention of an influenza pandemic.^13^

Influenza vaccination was shown to modify disease severity and is associated with decreased odds of hospital death and intensive care unit (ICU) confinement.^14^ Studies have shown that vaccination has a moderate preventive effect for influenza among the elderly and that it significantly decreases influenza outcomes including respiratory and cardiovascular complications.^15^ In addition, vaccination is associated with lower number of missed work during the flu season. It also protects health care workers who are in close contact with patients with influenza.^16^

The WHO recommends vaccination of children between 6 months and 5 years, and among elderly persons, pregnant women, those with chronic medical conditions and health care workers.^2,17^ Routine annual influenza vaccination has also been recommended by the CDC since 2010.^18^

Despite the global consensus about the importance of influenza vaccination in preventing influenza mortality and reducing unnecessary hospitalization, influenza vaccination coverage remains low in most parts of the world.^19^

Among US adults, less than half of the vaccination target has been achieved.^12,20^ In the Middle East, annual vaccination coverage is similarly low.^21^

In Saudi Arabia, there is a high coverage for routine childhood immunization. However voluntary immunization, as in the case of influenza vaccination is low to modest. The most recent estimates of vaccine coverage of which is at 44.5% among adults.^22^ Variations in vaccination rates between specific population groups exists as well. Studies among Saudi medical students and health care professionals showed that vaccination coverage to be 20.7% and 34.4%, respectively.^1,23^ Influenza vaccination among pregnant Saudi women remains to be low despite the reduction in respiratory illness that vaccination can provide.^11^

There are no officially published data on coverage targets from the Ministry of Health of Saudi Arabia. However, certain patient groups are identified as target population for annual influenza vaccination by the Ministry of Health. These include among others diabetics, asthmatics, patients with chronic obstructive pulmonary disease (COPD), patients with all types of chronic cardiac, renal or liver diseases, patients with neurological disorders, and immune deficiency patients whether congenital or acquired (due to cancer or steroids). The target groups also include individuals who are morbidly obese, women who are pregnant, children aged 6 months to 5 years, pediatric patients on long-term Aspirin therapy, persons older than 50 years, and all health care workers.^3^

Annual intensive influenza vaccination campaigns are carried out by government health workers from October to January. To increase access to free influenza vaccine, immunization drives are routinely conducted in schools, mosques, government offices, malls, and parks in addition to health facilities.

There are several researches done concerning influenza vaccination behavior. Studies have proposed that social determinants as well as intermediary factors such as behavioral beliefs, previous vaccination experience, perception on susceptibility, perceived health status and source of information affect vaccination behavior.^19^ Issues regarding vaccine safety and efficacy have similarly been proposed.^16^ There were studies that attribute low influenza vaccination in the Eastern Mediterranean Region to lack of awareness and knowledge while others point to inequities regarding vaccination policies.^19,24^ It is important to determine the actual role of these factors in the influenza vaccination of residents in Saudi Arabia.

This study sought to determine the influenza vaccination coverage among adult residents of Al Ahsa, Saudi Arabia; determine differences in vaccination status of various sociodemographic groups; and determine the effects of influenza vaccine awareness and confidence on vaccination status.

## Methods

### Study Population and Data Collection Strategy

This analytical cross-sectional study involved Saudi Arabian nationals aged 18 years and older at the time of data collection who were residing in Al Ahsa, Eastern Province. The sample size was determined using the following parameters: anticipated vaccination coverage of 44.53% based on the study of Alqahtani, *et al*. in 2017, margin of error of 5%, design effect of 2, and confidence level of 95%. The minimum sample size was calculated to be 759. This value was doubled to account for the possibility of sample size reduction due to incomplete responses to the main variable of interest.

**Figure 1.**
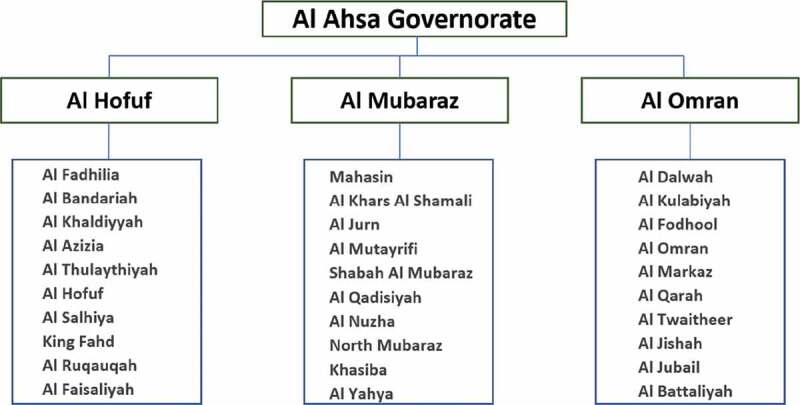
Flowchart of multi-stage sampling

Multi-stage sampling was utilized in the recruitment of the study participants. As seen in Figure 1, ten Primary Health Centers (PHC) were randomly selected from each of the three Public Health administrative sectors of Al Ahsa, Saudi Arabia namely: Al Hofuf, Al Mubaraz, and Al Omran. Each of the chosen PHCs were given 50 survey forms which were then accomplished by participants who were systematically selected by the PHC staff such that every third patient who came for consultation was invited to participate in the survey. The data collection took place between July and August 2019 with a response rate of 92%.

### Data Collection Instrument

The data collection instrument used in the study was a self-administered questionnaire developed by the investigators. It was composed of various questions pertaining to the sociodemographic characteristics of the study participants and ten questions related to influenza vaccine awareness and confidence. The questionnaire was translated into Arabic by an official translator and later translated back to English to ensure the contextual consistency of the questions. The tool was pretested among a sample of 30 individuals with similar characteristics as the study population.

### Definition of Major Study Variables

The influenza vaccination status of the respondent was determined through his/her response to the question *“Were you vaccinated against influenza in the past 12 months?*”. In this study, “vaccine awareness” pertained to a respondent’s awareness on the existence of a vaccine against influenza and similarly on its availability from any government health facility at no cost to the recipient. On the other hand, “vaccine confidence” was ascertained through a participant’s response to the following questions: “*Are you confident that influenza vaccine can prevent you from getting influenza?*”; “*Are you confident that the influenza vaccine can protect you against complications of influenza?„*; and *“Are you confident that the influenza vaccine is safe?”.*

### Data Processing and Statistical Analysis

All accomplished survey forms where checked for completeness of responses. Ten survey forms were excluded due to non-response to the main study variables or logical inconsistencies in the responses. All collected data from a total of 1,377 participants were encoded and processed in Epi Info® version 7.

Summary statistics for the sociodemographic characteristics of the respondents were generated. Various proportions were estimated using the Wilson method including influenza vaccination coverage and proportions of Al Ahsa residents who were aware of and confident on the efficacy and safety of influenza vaccine. The 95% confidence interval estimates were presented.

Logistic regression analyses were performed to determine the associations between vaccine awareness and confidence to the influenza vaccination status. Odds ratios and their corresponding 95% confidence interval estimates were calculated.

This study was reviewed and granted approval for implementation by the King Fahad Hospital Hofuf Research and CME Administration, with approval number KFHH RCA No. 07 18 2019 (dated 28/03/2019).

## Results

### Sociodemographic profile of respondents

The sociodemographic characteristics of study participants (n = 1377) are summarized in Table 1.

**Table 1.** Sociodemographic characteristics of study participants (n = 1377)

Socio-demographic factor	Frequency	Percent (%)
*Gender*		
Male	648	47.06
Female	729	52.94
*Age*		
18–27	247	17.94
28–37	628	45.61
38–47	305	22.15
48–58	137	9.95
58–67	45	3.27
68 and above	15	1.09
*Educational Attainment*		
Illiterate	2	0.15
Primary	69	5.01
Intermediate	131	9.51
High-school	344	24.98
College/University	774	56.21
Post-graduate	57	4.14
*Employment Statu*s		
Student/Intern	172	12.49
Employed – Government Sector	658	47.79
Employed – Private Sector	155	11.26
Retired	57	4.14
Unemployed	335	24.33

There was a slightly higher proportion of female respondents. The highest proportion of respondents belonged to the 28 to 37 age group. Majority have completed college-level education and were employed in the government sector.

### Proportion of vaccinated residents according to demographic groups

Among the 1377 study participants, 608 (44.15%) were vaccinated while 769 (55.85%) were not. The estimated influenza vaccination coverage was 44.15% (95% CI = 41.55–46.79%).

As seen in Table 2, 48 for every 100 males were vaccinated compared to 40 for every 100 females. By age group, the highest proportion of respondents who received immunization was in the 58 – 68 group with 56 for every 100 of them having been vaccinated in the past 12 months.

**Table 2.** Vaccination status of respondents according to sociodemographic characteristics

Sociodemographic factor	Vaccinated				Unvaccinated			
	Count	Proportion, %	95% CI		Count	Proportion, %	95% CI	
*Sex*								
Male	310	47.84	44.02	51.69	338	52.16	48.31	55.98
Female	298	40.88	37.37	44.49	431	59.12	55.51	62.63
*Age*								
18–27	90	36.44	30.43	42.78	157	63.56	57.22	69.57
28–37	271	43.15	39.33	47.06	357	56.85	52.94	60.67
38–47	142	46.56	41.04	52.16	163	53.44	47.84	58.96
48–57	74	54.01	45.30	62.56	63	45.99	37.44	54.70
58–67	25	55.56	40.00	70.36	20	44.44	29.64	60.00
68 and above	6	40.00	16.34	67.71	9	60.00	32.29	83.66
*Educational attainment*								
Below college	209	38.28	34.30	42.42	337	61.72	57.58	65.70
College and postgraduate	399	48.01	44.63	51.41	432	51.99	48.59	55.37
*Employment Status*								
Unemployed	189	33.51	29.74	37.51	375	66.49	62.49	70.26
Employed	419	51.54	48.10	54.96	394	48.46	45.04	51.90

When grouped by educational attainment, a higher proportion of influenza-vaccinated residents were seen among those who attained college degrees or higher. When classified by employment, a higher proportion of employed Al Ahsa residents received influenza vaccination.

### Awareness on influenza vaccination

As seen in Table 3, eight for every ten Al Ahsa residents were aware of the existence of a vaccine that provides protection against influenza. Furthermore, approximately 9 for every 10 Al Ahsa residents were aware that Saudi residents can avail of free influenza vaccination in government health facilities.

**Table 3.** Awareness on the availability of influenza vaccination services

Awareness Status	Counts	Proportion, %	95% CI	
*Availability of vaccine*				
Unaware	232	16.85	14.96	18.92
Aware	1145	83.15	81.08	85.04
*Free influenza vaccination*				
Unaware	166	12.06	10.44	13.88
Aware	1211	87.94	86.12	89.56

### Confidence on influenza vaccine efficacy and safety

It is estimated that six for every 10 residents were confident that the influenza vaccine can prevent influenza infection while roughly 7 for every 10 residents were confident that the said vaccine provides protection against the complications of influenza (Table 4).

**Table 4.** Confidence on influenza vaccination

Confidence Status	Counts	Proportion, %	95% CI	
*Prevention of influenza*				
Not confident	511	37.11	34.60	39.69
Confident	866	62.89	60.31	65.40
*Protection against complications of influenza*				
Not confident	432	31.37	28.98	33.87
Confident	945	68.63	66.13	71.02
*Safety of influenza vaccine*				
Not confident	402	29.19	26.85	31.65
Confident	975	70.81	68.35	73.15

Regarding influenza vaccine safety, it is estimated that 70.81% (95% CI = 68.35% – 73.15%) were confident that the influenza vaccine does not cause untoward effects on those who receive the vaccine.

### Crude associations between various sociodemographic characteristics, vaccine awareness, and vaccine confidence to vaccination status

Participants who were at least 36 years old were 30% more likely to have received influenza vaccination compared to younger Al Ahsa residents. Female residents were 25% less likely to have been vaccinated compared to male residents. Residents who had a higher educational attainment were 50% more likely to be vaccinated against influenza compared to residents who completed education below college level. Those who are currently employed were twice as likely to have received influenza vaccination in the past 12 months compared to those who were either unemployed or retired (Table 5).

**Table 5.** Association between sociodemographic variables and vaccination status

Variable	Crude OR (95% CI)	*p*-value
*Age* Older^a^ Younger^b^	1.3019 (1.0477–1.6177) 1.0000	.0173
*Gender* Female Male	0.7539 (0.6090–0.9332) 1.0000	.0095
*Educational attainment* High Low	1.4893 (1.1955–1.8553) 1.0000	.0004
*Employment status* Employed Unemployed	2.1100 (1.6891–2.6356) 1.0000	<.0001
*Awareness on vaccine availability* Yes No	2.9256 (2.1196–4.0380) 1.0000	<.0001
*Awareness on free vaccination* Yes No	7.3264 (4.4841–11.9701) 1.0000	<.0001
*Confidence that vaccine prevents influenza* Yes No	3.5043 (CI: 2.7592–4.4508) 1.0000	<.0001
*Confidence that vaccine protects against complications* Yes No	3.0554 (CI: 2.3816–3.9199) 1.0000	<.0001
*Confidence on vaccine safety* Yes No	4.3984 (3.3530–5.7699)	<.0001

^a^36.7 years and younger; ^b^older than 36.7 years

Al Ahsa residents who were aware of the availability of a vaccine against influenza were nearly 3 times more likely to be vaccinated compared to those who were not. Additionally, those who were aware that influenza vaccine is available for free in government health facilities were seven times more likely to be vaccinated compared to those who were not.

Al Ahsa residents who were confident that the influenza vaccine prevents influenza infection and those who were confident that it protects against influenza complications were 3.5 times and 3 times more likely to be vaccinated compared to their counterparts. Furthermore, those who were confident that the vaccine is safe were four times more likely to be vaccinated that those who are in doubt of its safety.

### Adjusted association between influenza vaccine awareness and immunization status

As seen in Table 6, Al Ahsa residents who were aware of the influenza vaccine were nearly 2.7 times more likely to be vaccinated when age, gender, educational attainment and employment status were held constant. A decline of 8.55% in the crude association was observed after adjusting for the said sociodemographic variables.

**Table 6.** Adjusted association of awareness on the availability of influenza vaccine and influenza vaccination status

Variable	aOR (95% CI)	*p*-value
*Awareness on vaccine availability* Yes No	2.6860 (1.9300–3.7381) 1.0000	<0.0001
*Age* Older^a^ Younger^b^	1.2080 (0.9590–1.5217) 1.0000	0.1085
*Gender* Female Male	0.9159 (0.7229–1.1605) 1.0000	0.4671
*Educational Attainment* High Low	1.1595 (0.9125–1.4733) 1.0000	0.2259
*Employment Status* Employed Unemployed	1.8277 (1.4240–2.3459) 1.0000	<0.0001

^a^36.7 years and younger; ^b^older than 36.7 years

On the other hand, residents who were aware that the influenza vaccine is available for free in government health facilities were six times more likely to be vaccinated compared to those who were not aware of free vaccination after controlling for the effects of sociodemographic variables. This represents a 17.09% decrease in the relative association between awareness of free vaccination and vaccination status (Table 7).

**Table 7.** Adjusted association of awareness on the availability of free vaccination service and vaccination status

Variable	aOR (95% CI)	*p*-value
*Awareness on free vaccination* Yes No	6.2626 (3.8070–10.3022) 1.0000	<0.0001
*Age* Older^a^ Younger^b^	1.1620 (0.9201–1.46761) 1.0000	0.2074
Gender Female Male	0.9473 (0.7456–1.2034) 1.0000	0.6572
*Educational Attainment* High Low	1.1208 (0.8795–1.4283) 1.0000	0.3565
*Employment Status* Employed Unemployed	1.7402 (1.3500–2.2431) 1.0000	<0.0001

^a^36.7 years and younger; ^b^older than 36.7 years

### Adjusted association between confidence on vaccine efficacy and safety and immunization status

Al Ahsa residents who were confident that the influenza vaccine can prevent influenza infection were nearly four times more likely to be vaccinated compared to those who were not confident when the effect of sociodemographic factors were controlled. This represents an increase of 3.58% in the relative association between confidence in vaccine efficacy and vaccination status (Table 8).

**Table 8.** Adjusted association of confidence on influenza vaccine efficacy for preventing influenza and vaccination status

Variable	aOR (95% CI)	*p*-value
*Confidence that vaccine prevents influenza* Yes No	3.6331 (2.8425–4.6436) 1.0000	<0.0001
*Age* Older^a^ Younger^b^	1.1670 (0.9206–1.4795) 1.0000	0.2018
*Gender* Female Male	1.0569 (0.8277–1.3497) 1.0000	0.6573
*Educational Attainment* High Low	1.2844 (1.0074–1.6377) 1.0000	0.0435
*Employment Status* Employed Unemployed	2.0954 (1.6190–2.7120) 1.0000	<0.0001

^a^36.7 years and younger; ^b^older than 36.7 years

As seen in Table 9, residents who were confident that the vaccine can prevent complications of influenza were three times more likely to have been immunized compared to those who were not confident when the effect of sociodemographic variables were controlled. This represents a 6.15% increase in the crude association between confidence on vaccine efficacy to prevent complications and vaccination status.

**Table 9.** Adjusted association of confidence on influenza vaccine efficacy to prevent complications and vaccination status

Variable	aOR (95% CI)	*p*-value
*Confidence that vaccine protects against complications* Yes No	3.2467 (2.5149–4.1914) 1.0000	<0.0001
*Age* Older^a^ Younger^b^	1.2241 (0.9681–1.5479) 1.0000	0.0912
*Gender* Female Male	0.9883 (0.7768–1.2573) 1.0000	0.9238
*Educational Attainment* High Low	1.2837 (1.0091–1.6330) 1.0000	0.0420
*Employment Status* Employed Unemployed	2.0792 (1.6117–2.6822) 1.0000	<0.0001

^a^36.7 years and younger; ^b^older than 36.7 years

Al Ahsa residents who were confident about the safety of the influenza vaccine were four and a half times more likely to be vaccinated compared who those who doubt the vaccine’s safety when sociodemographic variables were held constant. A 2.66% increase in the relative association between confidence on influenza vaccine safety and vaccination status was noted (Table 10).

**Table 10.** Adjusted association of confidence on influenza vaccine safety and vaccination status

Variable	aOR (95% CI)	p-value
*Confidence on vaccine safety* Yes No	4.5105 (3.4206–5.9477) 1.0000	<0.0001
*Age* Older^a^ Younger^b^	1.2326 (1.2326–1.5643) 1.0000	0.0855
*Gender* Female Male	1.0166 (0.7964–1.2977) 1.0000	0.8951
*Educational Attainment* High Low	1.2400 (0.9706–1.5843) 1.0000	0.0853
*Employment Status* Employed Unemployed	2.0476 (1.5816–2.6510) 1.0000	<0.0001

^a^36.7 years and younger; ^b^older than 36.7 years

## Discussion

The current study showed that less than half of the adult population of Al Ahsa were vaccinated against influenza in the past twelve months. Specifically, only four out of every ten Al Ahsa residents received influenza immunization in the past 12 months. This vaccination coverage is comparable to the findings of a 2016 study of a Saudi population reporting 44.53% coverage but higher than the 36.7% reported coverage in a study in Riyadh.^6,22^ However, the coverage is below the 70% target for Healthy People 2020 for noninstitutionalized individuals who are 18 years and above.^25^

The proportion of residents who received the influenza vaccine differed significantly between sexes, among age groups, between occupation groups, and between education groups. The influenza vaccination coverage for residents aged 50 and above was only 53.57%. The Saudi Arabian Ministry of Health targets and recommends individuals who are 50 and older for annual seasonal influenza vaccination.^3^

While majority of Al Ahsa residents were aware of the existence of the flu vaccine and its availability in the government health facilities in the Kingdom, a slightly lower proportion of the study population were actually confident about vaccine efficacy and safety. Barely 62% of the respondents were confident about its efficacy while 70% reported to be confident about its safety. Another study has reported that 84.51% of Saudis believe that the influenza vaccine is safe and effective.^22^

This study has shown that both vaccine awareness and vaccine confidence affect the likelihood of being immunized. Awareness of the availability of influenza vaccine increases the likelihood of an individual to voluntarily submit himself for vaccination. Moreover, the likelihood increased by two and a half times with an awareness that the said vaccine was available free of charge.

Confidence on efficacy and safety both increased the likelihood of being immunized. Confidence on the vaccine’s efficacy in preventing influenza or its complications more than triples the likelihood of being immunized. Confidence on vaccine safety increases the likelihood of being immunized four-fold. Therefore, confidence of vaccine safety was a more important determinant of vaccination status compared to confidence on efficacy among residents of Al Ahsa.

Efforts in building the awareness of people on the availability of free influenza vaccine and confidence of people on the efficacy and safety of the vaccine cannot be overemphasized. Information dissemination regarding the availability of the influenza vaccine in government health facilities must be enhanced. Innovative educational interventions focusing on improving the confidence of residents on the efficacy and safety of the influenza vaccine such as aggressive print, radio, television and social media campaigns must be implemented.

There were several limitations to this study. There is a high tendency of misclassification as the responses were self-reported based on respondents’ recollection of previous influenza immunization. The possibility of information bias cannot be discounted for awareness and confidence as they reflect only the perception of the respondents. Furthermore, the health-seeking characteristics of the study participants may not completely represent the general population of Al Ahsa. Residents who usually seek medical services in private health facilities and those who did not visit the PHC during the period of data collection may not have been equally represented. Those who participated in the study were more likely familiar with and supportive of the health services provided free of charge in the PHC and other government health facilities. The differential selection probabilities might have over-estimated the measures of vaccine awareness and confidence.

Seasonal influenza coverage in Al Ahsa remains low despite the availability of free immunization in the government health facilities. Most of Al Ahsa residents are aware of the availability of influenza vaccination. While vaccine coverage differs between different demographic groups, influenza vaccination is associated with awareness of vaccine availability and with confidence in both vaccine efficacy and safety. Vaccine awareness and vaccine confidence must be jointly addressed to raise the influenza vaccination coverage in Al Ahsa.

## Supplementary Material

Supplemental Material

## References

[1] Abalkhail M, Alzahrany M, Alghamdi K, Alsoliman M, Alzahrani M, Almosned B, Gosadi I, Tharkar S. Uptake of influenza vaccination, awareness and its associated barriers among medical students of a University Hospital in Central Saudi Arabia. J Infect Public Health. 2017 9 1;10(5):644–48. doi:10.1016/j.jiph.2017.05.001. 28545902

[2] World Health Organization. Influenza (Seasonal). Geneva: World Health Organization;2018 11. [accessed 2019 313. https://www.who.int/news-room/fact-sheets/detail/influenza-seasonal.

[3] Ministry of Health. Seasonal Influenza Vaccination. Riyadh (KSA): Ministry of Health; accessed 2019 313. https://www.moh.gov.sa/en/Flu/Pages/About.aspx.

[4] Centers for Disease Control and Prevention. Seasonal flu deaths estimate increases worldwide. Altanta (CDC); 2017 12. accessed 2019 313 https://www.cdc.gov/media/releases/2017/p1213-flu-death-estimate.html

[5] Centers for Disease Control and Prevention. 2018-2019 U.S. Flu Season: preliminary Burden Estimates; [accessed 2019 313, 2019]. https://www.cdc.gov/flu/about/burden/preliminary-in-season-estimates.htm

[6] Sagor K, AlAteeq M. Beliefs, attitudes and barriers associated with the uptake of the seasonal influenza vaccine among patients visiting primary healthcare clinics. Saudi Med J. 2018 7;39(7):690. doi:10.15537/2Fsmj.2018.7.22293. 29968892 PMC6146252

[7] World Health Organization. International travel and health: seasonal Influenza. Geneva: World Health Organization; [accessed 2019 313. https://www.who.int/ith/diseases/influenza_seasonal/en/.

[8] World Health Organization. A manual for estimating disease burden associated with seasonal influenza. Geneva: World Health Organization; 2015. [accessed 2019 313. https://apps.who.int/iris/bitstream/handle/10665/178801/9789241549301_eng.pdf;jsessionid=8D33D9406ACEE32C54D66E770FE599ED?sequence=1.

[9] Schmid P, Rauber D, Betsch C, Lidolt G, Denker M. Barriers of influenza vaccination intention and behavior–a systematic review of influenza vaccine hesitancy, 2005–2016. PloS One. 2017 1 26;12(1):e0170550. doi:10.1371/journal.pone.0170550. 28125629 PMC5268454

[10] World Health Organization. Up to 650 000 people die of respiratory diseases linked to seasonal flu each year. Geneva: World Health Organization;2017 12. [accessed 2019 313. https://www.who.int/news-room/detail/14-12-2017-up-to-650-000-people-die-of-respiratory-diseases-linked-to-seasonal-flu-each-year

[11] Mayet A, Al-Shaikh G, Al-Mandeel H, Alsaleh N, Hamad A. Knowledge, attitudes, beliefs, and barriers associated with the uptake of influenza vaccine among pregnant women. Saudi Pharm J. 2017 1 1;25(1):76–82. doi:10.1016/j.jsps.2015.12.001. 28223865 PMC5310150

[12] Mossad S. Influenza update 2018–2019: 100 years after the great pandemic. Cleve Clin J Med. 2018 11 1;85(11):861–69. doi:10.3949/ccjm.85a.18095. 30395523

[13] Yaqub O, Castle-Clarke S, Sevdalis N, Chataway J. Attitudes to vaccination: a critical review. Soc Sci Med. 2014 7 1;112:1–1. doi:10.1016/j.socscimed.2014.04.018. 24788111

[14] Arriola C, Garg S, Anderson E, Ryan P, George A, Zansky S, Bennett N, Reingold A, Bargsten M, Miller L, et al. Influenza vaccination modifies disease severity among community-dwelling adults hospitalized with influenza. Clin Infect Dis. 2017 10 15;65(8):1289–97. doi:10.1093/cid/cix468. 28525597 PMC5718038

[15] Kan T, Zhang J. Factors influencing seasonal influenza vaccination behaviour among elderly people: a systematic review. Public Health. 2018 3 1;156:67–78. doi:10.1016/j.puhe.2017.12.007. 29408191 PMC7111770

[16] Mytton O, O’moore E, Sparkes T, Baxi R, Abid M. Knowledge, attitudes and beliefs of health care workers towards influenza vaccination. Occup Med (Lond). 2013 4 1;63(3):189–95. doi:10.1093/occmed/kqt002. 23447033

[17] Rondy M, El Omeiri N, Thompson MG, Levêque A, Moren A, Sullivan S. Effectiveness of influenza vaccines in preventing severe influenza illness among adults: A systematic review and meta-analysis of test-negative design case-control studies. J Infect. 2017 11 1;75(5):381–94. doi:10.1016/j.jinf.2017.09.010. 28935236 PMC5912669

[18] Centers for Disease Control and Prevention. Prevention and control of seasonal influenza with vaccines: recommendations of the advisory committee on immunization practices— united states, 2018–19 influenza season. U.S. department of health and human services centers for disease control and prevention. Morbidity and Mortality Weekly Report. Recommendations and Reports/Vol. 67/No. 3. 10.15585/mmwr.rr6703a1 PMC6107316 30141464

[19] Nagata J, Hernández-Ramos I, Kurup A, Albrecht D, Vivas-Torrealba C, Franco-Paredes C. Social determinants of health and seasonal influenza vaccination in adults≥ 65 years: a systematic review of qualitative and quantitative data. BMC Public Health. 2013 12;13(1):1–25. doi:10.1186/1471-2458-13-388. 23617788 PMC3667118

[20] Rogers C, Bahr K, Benjamin S. Attitudes and barriers associated with seasonal influenza vaccination uptake among public health students; a cross-sectional study. BMC Public Health. 2018 12 1;18(1):1131. doi:10.1186/s12889-018-6041-1. 30236092 PMC6148773

[21] Al Awaidy S, Althaqafi A, Dbaibo G, East M, Network NA. A snapshot of influenza surveillance, vaccine recommendations, and vaccine access, drivers, and barriers in selected Middle Eastern and North African Countries. Oman Med J. 2018 7;33(4):283. 30038727 10.5001/omj.2018.54 PMC6047181

[22] Alqahtani A, Althobaity H, Al Aboud D, Abdel-Moneim A. Knowledge and attitudes of Saudi populations regarding seasonal influenza vaccination. J Infect Public Health. 2017 11 1;10(6):897–900. doi:10.1016/j.jiph.2017.03.011. 28473261

[23] Rehmani R, Memon J. Knowledge, attitudes and beliefs regarding influenza vaccination among healthcare workers in a Saudi hospital. Vaccine. 2010 6 11;28(26):4283–87. doi:10.1016/j.vaccine.2010.04.031. 20441803

[24] Zaraket H, Melhem N, Malik M, Khan WM, Dbaibo G, Abubakar A. Review of seasonal influenza vaccination in the Eastern Mediterranean Region: policies, use and barriers. J Infect Public Health. 2020 3 4;13(3):377–84. doi:10.1016/j.jiph.2020.02.029. 32146138

[25] Office Disease Prevention and Health Promotion. Immunization and Infectious Diseases IDD 12.12. Washington: Healthy People.gov; 2020 2 25. https://www.healthypeople.gov/2020/topics-objectives/topic/immunization-and-infectious-diseases/objectives.

