# Supplementary material for: Association of vaccine awareness and confidence on the influenza vaccination status of Al Ahsa, Saudi Arabia residents
Source: Hum Vaccin Immunother. 2021 Jan 30;17(7):2190–6. doi: 10.1080/21645515.2020.1855954 (PMC8189070; doi:10.1080/21645515.2020.1855954)
Supplement: Supplemental Material [file KHVI_A_1855954_SM2341.docx]

Dear Al-Ahsa Residents:

You are invited to participate in a research study titled “Awareness, Confidence and Hesitancy on Influenza Vaccination Among Al Ahsa Residents”. This study is being conducted by Public Health specialists and health care workers of King Faisal University and the Ministry of Health. The purpose of this study is to determine the influenza immunization prevalence and explore the various factors affecting adult immunization attitudes and practices among residents of Al-Ahsa.

In this study, you will be asked to complete this questionnaire. Your participation in this study is voluntary and you are free to withdraw your participation from this study at any time. The survey should take only 3 minutes to complete.

The Institutional Review Board of the Ministry of Health – King Fahad Hospital Hofuf, has approved this survey (KFHH RCA No: 07-18-2019). There are no risks associated with participating in this study. The survey collects no identifying information of any respondent. All of the responses in the survey will be recorded anonymously.

Information collected in this study will contribute to the efficient delivery of health services in Al Ahsa, particularly in the aspect of disease prevention.

If you have any questions regarding the survey or this research project in general, please contact Yasser Taher Al Hassan at email [yasserphkfu@gmail.com](mailto:yasserphkfu@gmail.com) or mobile number 0549985884.

By completing and submitting this survey, you are indicating your consent to participate in the study.

Your participation is appreciated.

Very truly yours,

Yasser Taher Al-Hassan

Eduardo L. Fabella

Edric D. Estrella

Fatimah Hassan Al Saleh

Hassan Abdulfatah Al Ramadan

**Awareness, Confidence and Hesitancy on Influenza Vaccination Among Al Ahsa Residents**

| Year of Birth: ______________________ | Residence  District: _________________  City: ____________________ |
| --- | --- |
| Gender   - Male - Female   Are you currently pregnant?   - - Yes   - No | Marital Status   - Single - Married - Widow/Widower - Divorced |
| Profession:   - Student/Intern - Government employee - Private employee - Unemployed | Field of Employment   - Health - Non –Health - Not applicable *(currently unemployed)* |
| Education Attained   - Primary - Intermediate - High School - University - Post Graduate | Do you have any of the following diseases?   \|  \| Yes \| No \| \| --- \| --- \| --- \| \| Diabetes \|  \|  \| \| Hypertension \|  \|  \| \| Cancer \|  \|  \| \| Asthma \|  \|  \| |

1. Did you get influenza in the last 12 months?

- Yes
- No

2. Did you have to be confined in a hospital during your recent bout of influenza?

- Yes
  - Number of days in the hospital _______
  - Number of days absent from school, work _____________
- No

3. Are you aware that there is a vaccine that can protect you against influenza?

- Yes
- No

4. Are you aware that the influenza vaccine is available for free from government health facilities (health centers/hospital)?

- Yes
- No

5. Are you confident that influenza vaccine can prevent you from getting influenza?

- Yes
- No

6. Are you confident that the influenza vaccine can protect you against complications of influenza?

- Yes
- No

7. Are you confident that the influenza vaccine is safe?

- Yes
- No

8. Were you vaccinated against influenza in the past 12 months?

- Yes
- No

9. Are you planning to get influenza immunization in the next 12 months?

- Yes. Thank you for completing the survey.
- No

10. What is your reason for not getting vaccinated against influenza?

*Select all that apply.*

- Going for vaccination interferes with my work/school schedule.
- I read negative information about vaccines from social media (Facebook, Twitter, Instagram).
- I am healthy. I do not need it.
- I think it is painful.
- My family doctor did not advise me to get vaccinated.
- My friends advised me not to get vaccinated.
- I am allergic to flu vaccine.
- I did not receive information about flu vaccination.
- It was not available when I went to the health center.
- My family advised me not to get vaccinated.
- Flu vaccination is not required by my company/school.
- The health center/clinic is far from my home/place of work.
- I am worried about side effects.

**THANK YOU FOR YOUR PARTICIPATION**
